# Evaluation of transarterial chemoembolization refractoriness in patients with hepatocellular carcinoma

**DOI:** 10.1371/journal.pone.0229696

**Published:** 2020-03-04

**Authors:** Jonggi Choi, Danbi Lee, Ju Hyun Shim, Kang Mo Kim, Young-Suk Lim, Yung Sang Lee, Han Chu Lee

**Affiliations:** Department of Gastroenterology, Liver Center, Asan Medical Center, University of Ulsan College of Medicine, Seoul, Republic of Korea; Cincinnati Children’s Hospital Medical Center, UNITED STATES

## Abstract

**Background & aim:**

In clinical practice, transarterial chemoembolization (TACE) has been widely used for the treatment of hepatocellular carcinoma (HCC) beyond as well as within guideline recommendations. Here we aimed to verify whether two consecutive non-responses could be an optimal criterion for creating a rule to stop TACE being performed on these patients.

**Methods:**

This study evaluated 200 patients with HCC beyond the Milan criteria, initially treated with TACE. TACE response was determined using the mRECIST criteria via dynamic CT or MRI. Median follow-up duration was 23.9 months.

**Results:**

Within the 200 patients analyzed, 183 (91.5%) were male, with a total median age of 59.8 years. The mean size of the largest tumor was 6.8 cm, with 80 (40.0%) patients with ≥4 tumors. After the first TACE procedure, complete response, partial response, stable disease, or progressive disease were observed in 48 (24.0%), 87 (43.5%), 59 (29.5%) and 6 (3.0%) of patients, respectively. 45 (22.5%) patients showed no objective response (OR) following two consecutive TACE sessions. Of these, 28 received a subsequent TACE, with a 10.7% OR rate. Patients without OR showed poorer survival when compared to patients who achieved OR after repeated TACE. Multivariable analysis showed that size of the largest tumor >5cm and high alpha-fetoprotein of >200 ng/mL were significant factors associated with failure of OR to two consecutive TACE sessions.

**Conclusion:**

Patients showing no OR to two consecutive TACE sessions will present a poor OR to subsequent TACE procedures. Early transition to systemic therapy may be advocated in such cases.

## Introduction

Transarterial chemoembolization (TACE) is recommended as a first-line therapy for patients with intermediate-stage hepatocellular carcinoma (HCC) by most international guidelines including the Barcelona Clinic Liver Cancer (BCLC) staging system, although it has been applied to even broader indications in the clinic.[1–4] Unlike indications for TACE by current treatment guidelines, there is no widely accepted rule for stopping TACE. Previous reports showed different criteria for stopping TACE depending on the clinical trial.[3–5] Generally, repeated TACE is not recommended in patients who experience untreatable disease progression including extrahepatic metastasis, vascular invasion, intrahepatic progression associated with impaired liver function and extensive liver involvement.[6] Recently, in light of the success reported for several systemic agents, there has been an attempt to switch TACE to systemic therapy earlier than recommended by traditional criteria, especially in patients who are not considered to benefit from additional TACE treatment. Some guidelines have defined these TACE-refractory cases as patients with no objective responses (OR) after two or more sessions of TACE in addition to the conventional criteria of appearance of vascular invasion or extrahepatic spread, and therefore have recommended switching TACE to other treatment modalities.[6, 7] Despite these reports, this new criterion is not yet evidence-based, but consensus-based. Few studies have systematically analyzed the benefit of additional TACE according to the tumor’s response to previous TACE treatment.

Therefore, the aim of this study was to evaluate the response to TACE according to previous responders, and attempt at clarifying whether two consecutive non-responses could be an optimal criterion to introduce a guideline to stop further TACE treatment for these patients.

## Patients and methods

### Study population

We retrospectively collected 200 consecutive patients who were treated with TACE as an initial therapy for HCC outside the Milan criteria at the Asan Medical Center, Seoul, Republic of Korea, between January 2012 and July 2018. All anonymized data were obtained from the electronic medical records of Asan Medical Center (data accessed from November 2018 to October 2019) HCC was diagnosed by histology or typical radiology findings according to international guidelines.[6, 8, 9] Selected patients were all treatment-naïve and had multiple tumors with >3cm as the maximal diameter (n = 162) or a single HCC >5 cm in size which was not indicated for curative surgery (n = 38) and had Child-Pugh-Turcotte score of 7 or lower. All patients had at least one target lesion measuring ≥1 cm in diameter at baseline with features typical of HCC, including arterial enhancement followed by washout during the portal venous phase as visualized by dynamic computed tomography (CT) or magnetic resonance imaging (MRI). Tumors with invasion to the major portal vein or extrahepatic metastasis were excluded. The infiltrative HCC type was also excluded due to difficulty in taking accurate repeated measurements to assess tumor responses.[10] This study was approved and was exempted from obtaining consent by the institutional review board of the Asan Medical Center, Seoul, Republic of Korea. (IRB Number. 2018–1455)

### TACE procedure

The TACE procedure was performed in our hospital as previously described.[11] Briefly, both superior mesenteric and common hepatic arteriography were performed to assess overall anatomy, tumor burden and portal vein patency. After selective catheterization of the feeding artery using a microcatheter (diameter 1.8, 2.0, 2.2 or 2.4 Fr), cisplatin (2 mg/kg body weight in distilled water; 4 mL/kg) or adriamycin (50 mg) was infused into the segmental, lobar, or proper hepatic artery, according to the location and volume of the tumor, for 15 minutes. Before the 15 minutes cisplatin or adriamycin infusion, some cisplatin or adriamycin was set aside to be mixed at a 1:1 ratio in an emulsion of iodized oil (lipiodol; Guerbet, Roissy, France), which was infused into the segmental, subsegmental, or more peripheral level feeding artery, followed by embolization with Gelfoam slurry (Upjohn, Kalamazoo, MI) until a near stasis of arterial flow.[12, 13] The dose of iodized oil depended on the tumor size and ranged 3–20 mL. The emulsion of iodized oil and cisplatin or adriamycin was slowly injected until the accumulation of the emulsion in the tumor and the visualization of the portal vein branches near the tumor.[12–15] If there was a significant arterioportal shunt, embolization with a Gelfoam slurry was first performed to occlude the shunt, after which the iodized oil/cisplatin or adriamycin emulsion was infused and embolization with a Gelfoam slurry was performed.[12–14] This embolization was performed to minimize the ischemic toxicity of the non-targeted normal liver parenchyma and to minimize vascular complications due to the repeated endovascular procedures.[16] On-demand subsequent TACE was performed 6–10 weeks following previous TACE if residual viable tumor was obvious upon dynamic CT or MRI unless there was evidence of major vascular invasion, extrahepatic spread or deterioration of liver functions as indicated by a Child-Pugh-Turcotte score higher than 8. If compact lipiodolization without arterial enhancement at previous tumor sites was evident after the first or repeated TACE, then a follow-up by dynamic CT or MRI with measurements of serum alpha-fetoprotein (AFP) levels was performed in 2 or 3 month intervals at the physicians’ discretion. The median follow-up duration in the present study defined as the time between the first TACE and death or last follow-up date was of 23.9 months (interquartile range [IQR] 15.1–28.3 months).

### Assessment of response to TACE

All patients were examined using dynamic CT or MRI within 1–2 months after each TACE procedure to assess the radiological response of the tumor to treatment. Serum AFP levels, liver functions were measured simultaneously. Tumor response was assessed using the unidimensional modified Response Evaluation Criteria in Solid Tumors (mRECIST) criteria, according to four response categories: complete response (CR), partial response (PR), stable disease (SD), or progressive disease (PD).[10] All responses were based on the arterial-dominant phase of the dynamic CT or MRI at baseline, and upon follow-up in every session.[10] Iodized oil deposits in the tumors were rated as necrotic areas in keeping with previous studies.[11, 17] An objective response rate (ORR) was calculated as the proportion of patients showing a CR or PR.

#### Statistical analysis

Quantitative variables were compared using a Student’s t tests and qualitative variables using the chi-square test or Fisher’s exact test, as appropriate. Overall survival (OS) was defined as the time from the date of the first TACE procedure to date of death from any cause or the last date of confirmed survival. The OS rate was estimated using the Kaplan-Meier method. A binary logistic regression analysis was used to assess the significant factors associated with no objective response to two sessions of TACE, with multivariate adjustment for confounding variables that had shown significance in the univariate analysis. A p value <0.05 was considered statistically significant. The R-program was used for all statistical analyses.

## Results

### Patient characteristics

The study population and its characteristics are described in Table 1. From a total of 200 patients, 182 (91.0%) were male and the mean age was 59.7 years. Hepatitis B viral infection (68.5%) was the main cause of the underlying liver disease. The mean size of the largest tumor was 6.8 cm, with 81 (40.5%) patients presenting with four or more tumors. All patients included in this study were treatment-naïve with a median Child-Pugh-Turcotte score of 6 (IQR: 5–6).

**Table 1:** Baseline characteristics of the study population.

Characteristics	N = 200
Age, year, mean ± SD	59.7 ± 9.2
Sex, male	182 (91.0%)
Etiologies of liver disease	
Alcohol	24 (12.0%)
HBV	137 (68.5%)
HCV	17 (8.5%)
Others	22 (11.0%)
Laboratory findings	
Platelet count^§^, *x1000/mm*^*3*^	163.0 [116.5–206.5]
Serum albumin^§^, *g/dL*	3.5 [3.2–3.8]
Bilirubin^§^, *mg/dL*	0.6 [0.5–0.9]
Prothrombin time^§^, *%*	86.1% [77.3%-95.1%]
INR^§^	1.1 [1.0–1.1]
ALT^§^, *IU/L*	35 [24–53]
Child-Pugh score	
5–6	178 (89.0%)
7–8	22 (11.0%)
MELD score	7 [7–9]
Number of tumors	
1	39 (19.5%)
2	49 (24.5%)
3	31 (15.5%)
≥4	81 (40.5%)
Tumor size, mean ± SD, *cm*	
Largest	6.8 ± 4.3
Second largest^†^	2.6 ± 2.3
Third largest^‡^	1.5 ± 0.8
Outside Milan criteria	200 (100.0%)
Up to seven criteria	
Within up-to-seven criteria	48 (24.0%)
Beyond up-to-seven criteria	152 (76.0%)
Alpha-fetoprotein, ng/mL	
Median	49.5 [10.0–508.0]
≤200	66 (33.0%)
>200	134 (67.0%)

^†^161 patients with multiple tumors

^‡^112 patients with number of tumors >2

^§^Numbers are presented as median and numbers in brackets are the interquartile ranges

HBV: hepatitis B virus, HCV: hepatitis C virus, SD: standard deviation

### Overall response to the first and second TACE

**Fig 1:**
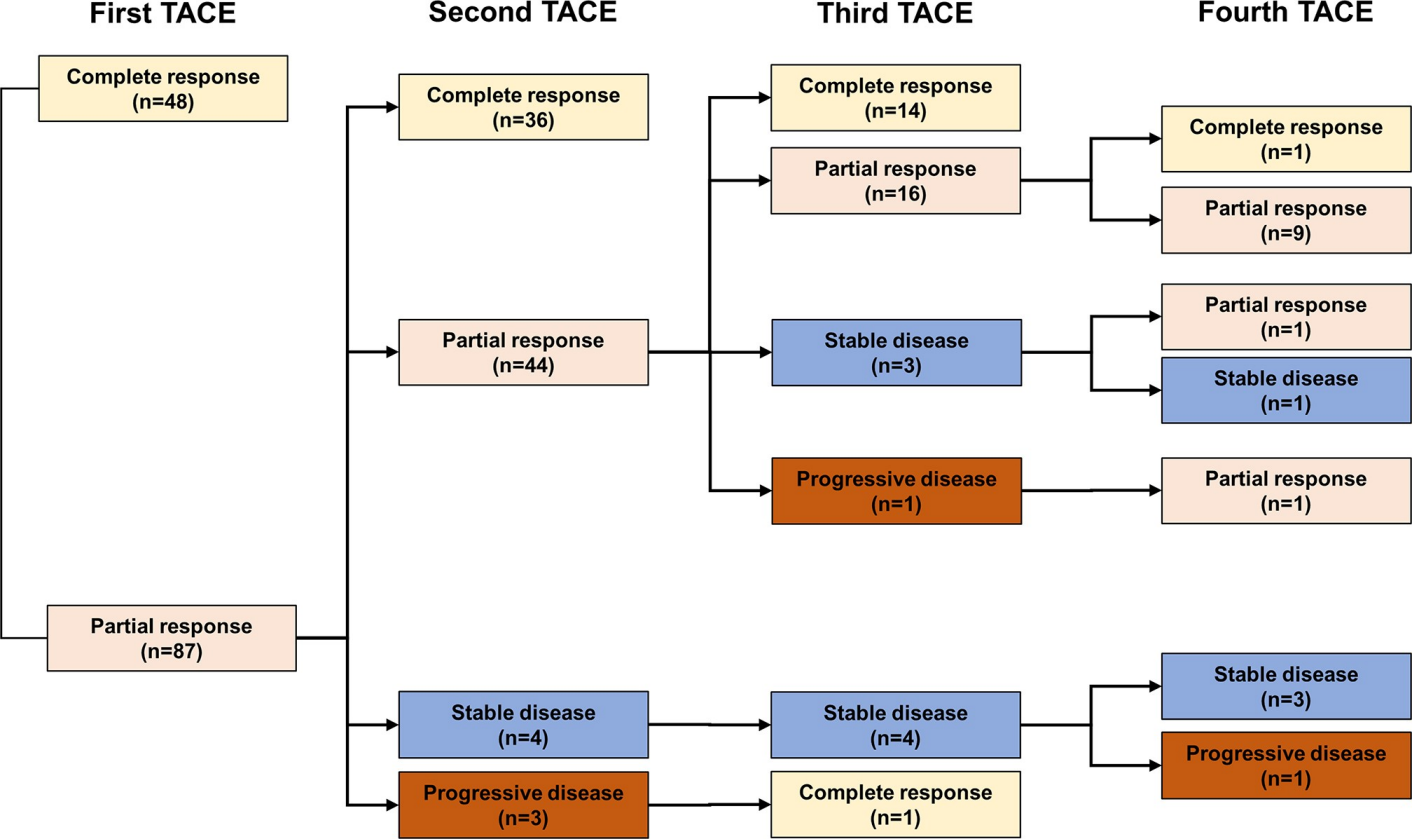
Responses to subsequent TACE in patients showing an objective response to the first TACE.

**Fig 2:**
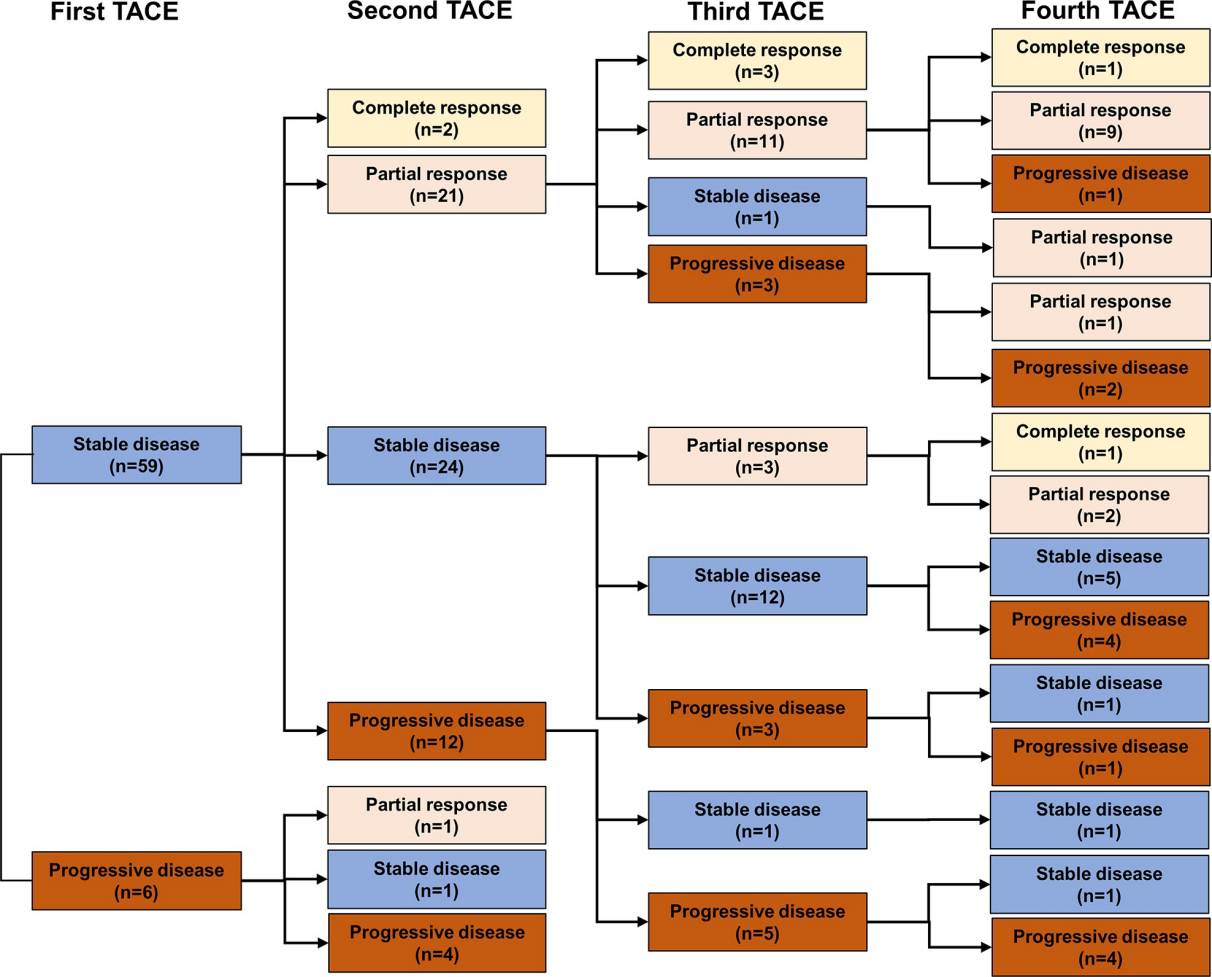
Responses to subsequent TACE in patients without objective response to the first TACE.

After the first TACE procedure, CR, PR, SD, and PD were observed in 48 (24.0%), 87 (43.5%), 59 (29.5%) and 6 (3.0%) of patients, respectively (Fig 1). Of the 87 patients achieving PR after the first TACE session, 36 (41.4%), 44 (50.6%), 4 (4.6%), and 3 (3.4%) showed CR, PR, SD, and PD after the second TACE session, respectively. Of the 65 patients who did not show an OR after the first TACE procedure (SD: 59 and PD: 6), only 2 (3.1%) achieved CR and 22 (33.8%) achieved PR in response to the second TACE session, yielding an ORR of 36.9% following two TACE sessions (Fig 2).

### Response to third and fourth TACE according to the response to first and second TACE

Of 44 patients (PR-PR), 34 received a third session of TACE and 14 (41.2%) showed CR and 16 (47.1%) patient showed PR, with an ORR of 88.3%, whereas the rest of 10 patients (PR-PR) were treated with local ablation therapy, external-beam radiation therapy, and best supportive care in 8, 1, and 1 patient, respectively. Among 7 patients (PR-SD or PR-PD), 5 patients underwent a third session of TACE, with only 1 (20.0%) patients showing an OR to the third TACE (Fig 2).

Of 21 patients (SD-PR), 18 underwent TACE three times consecutively. In this group, 3 (16.7%) achieved CR, 11 (61.1%) achieved PR, and 4 (22.2%) did not achieve an OR.

Among 24 patients (SD-SD), 18 patients received a third session of TACE, only with 3 (16.7%) showing PR. Of 12 patients (SD-PD), 6 underwent TACE for a third time. However, for these patients no OR was observed, despite the repeated TACE sessions (Fig 2).

Regarding the fourth TACE session, of 11 (SD-PR-PR) maintained OR at the fourth TACE session except for 1 patient who showed PD due to a new intrahepatic lesion. Among 15 patients (SD-SD-SD or SD-SD-PD), 11 of those received a fourth TACE session, with a 0% of ORR. Similarly, 6 (SD-PD-SD or SD-PD-PD) without OR in previous three session of TACE did not achieve an OR at the fourth TACE session.

### Response to subsequent TACE according to responses to previous TACE

Of 55 patients [PR-PR: 44 and SD-PR-PR: 11] who showed a PR consecutively at any point after repeated TACE, 15 (27.3%) achieved CR and 25 (45.5%) maintained PR at the following TACE session, with an ORR of 72.7%.

Collectively, 45 showed no OR twice consecutively at any time during repeated TACE, and these included 24 (SD-SD), 12 (SD-PD), 1 (PD-SD), 4 (PD-PD), and 4 (PR-SD-SD). Of these 45 patients, 28 received subsequent TACE [18 (SD-SD), 6 (SD-PD), and 4 (PR-SD-SD)] albeit without OR to previous two-consecutive TACE sessions. In this group, only 3 patients (10.7%) showed PR at the next TACE session. The rest of 17 patients who did not receive further TACE sessions, 6 started treatment with sorafenib, 4 received TACE with external beam radiation to malignant portal vein thrombosis, one underwent palliative resection, and one received other locoregional treatment. Five patients did not receive further treatment due to poor liver function or performance status.

#### Factors that predict a poor response to repeated TACE

Table 2 summarized factors predictive of poor response to repeated TACE. In multivariable analysis, the size of the largest tumor >5cm (Adjusted odds ratio [AOR]: 3.74, 95% CI: 1.57–8.90, P = 0.003) and AFP >200 ng/mL (AOR: 3.11, 95% CI: 1.40–6.91, P = 0.005) was associated with poor responses to repeated TACE (Table 2).

**Table 2:** Univariate and multivariable analysis of factors associated with no objective response to two sessions of TACE.

Variables	Univariate			Multivariable		
	OR	95% CI	*P* value	AOR	95% CI	*P* value
Gender, male	2.51	0.56–11.36	0.23			
Age, per 1-year increase	0.98	0.95–1.02	0.29			
Etiologies						
Alcohol	1	Reference		1	Reference	
HBV	0.42	0.17–1.06	0.06	0.30	0.11–0.84	0.02
HCV	0.22	0.04–1.21	0.08	0.38	0.07–1.77	0.24
Others	0.78	0.22–2.63	0.69	0.65	0.17–2.38	0.51
Largest tumor size						
≤ 5cm	1	Reference		1	Reference	
> 5cm	4.11	1.86–9.09	<0.001	3.74	1.57–8.90	0.003
Tumor number						
≤ 3	1	Reference				
> 3	1.51	0.78–2.92	0.23			
Alpha-fetoprotein, ng/mL						
≤ 200	1	Reference		1	Reference	
> 200	3.23	1.63–6.40	<0.001	3.11	1.40–6.91	0.005
Up-to-seven criteria^†^						
Within up-to-seven criteria	1	Reference				
Beyond up-to-seven criteria	5.81	1.71–19.69	0.005			

^†^Variable was not included to multivariable analysis due to collinearity

AOR: adjusted odds ratio, CI: confidence interval, HBV: hepatitis B virus, HCV: hepatitis C virus, OR: odds ratio, TACE: transarterial chemoembolization

### Changes in liver function in patients who showed no response to repeated TACE

**Fig 3:**
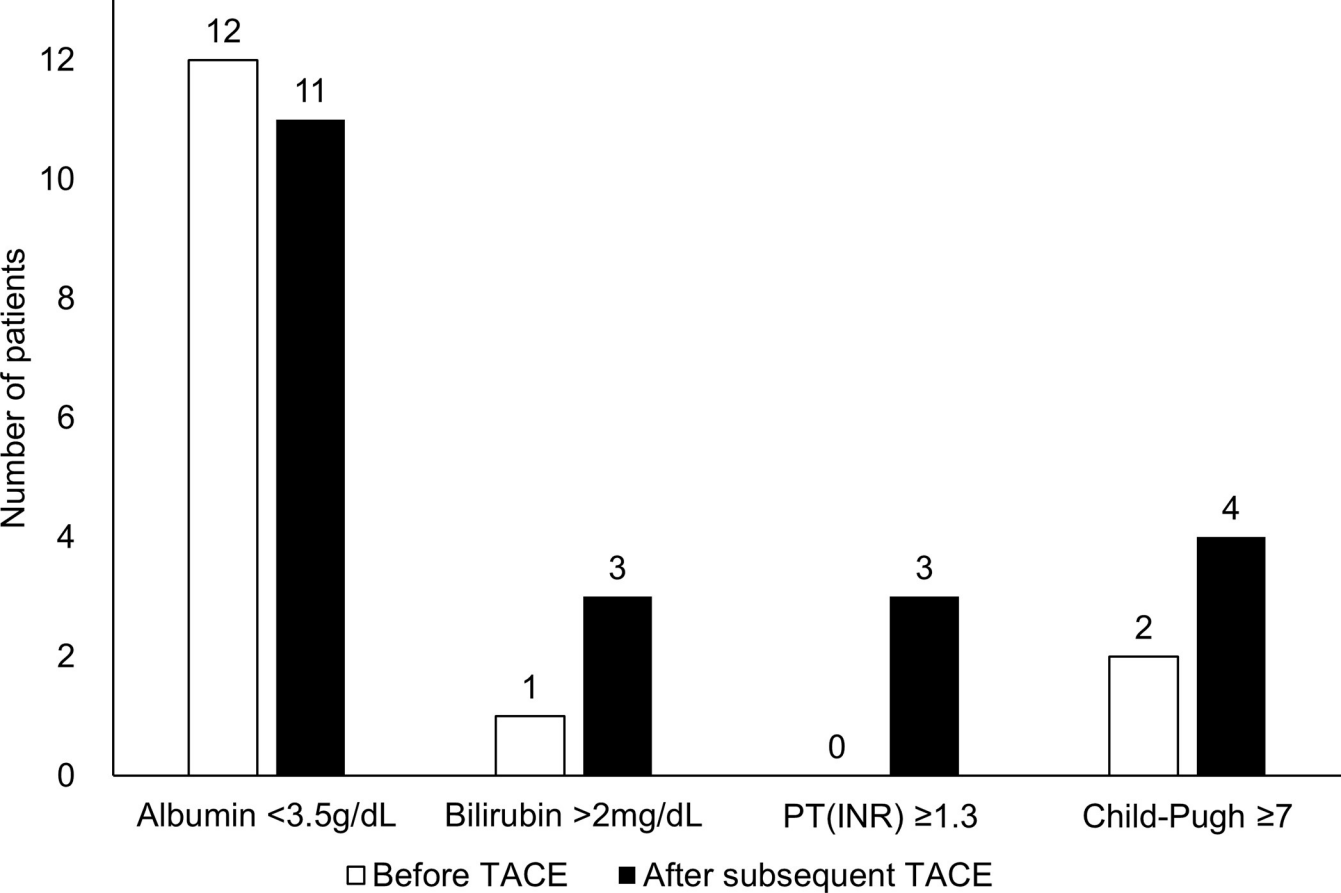
Of 28 patients who underwent subsequent TACE session, number of patients who deteriorate liver function after showing no objective response to previous TACE sessions.

Of the 45 patients who showed no OR consecutively to two previous TACE sessions, 3, 5, and 20 patients received subsequent TACE one, two, or three times, respectively. Of those 28 patients, no significant differences in serum albumin were evident in the period of time of subsequent TACE treatment (Fig 3). By contrast, 3 patients had hyperbilirubinemia (> 2 mg/dL) and 4 had Child-Pugh-Turcotte class B (≥ 7) during the same period. No patients showed prolonged prothrombin time (INR >1.3) at the time when a no OR was recorded, whereas 3 patients did show a prolonged PT (INR>1.3) after receiving subsequent TACE (Fig 3).

### Stage progression and post progression treatment

Of the 45 patients who did not show an OR consecutively after the two sessions of TACE, 28 (62.2%) progressed to advanced-stage HCC during the observation period, with only 12 (26.7%) being switched to sorafenib treatment.

### Survival according to treatment response

**Fig 4:**
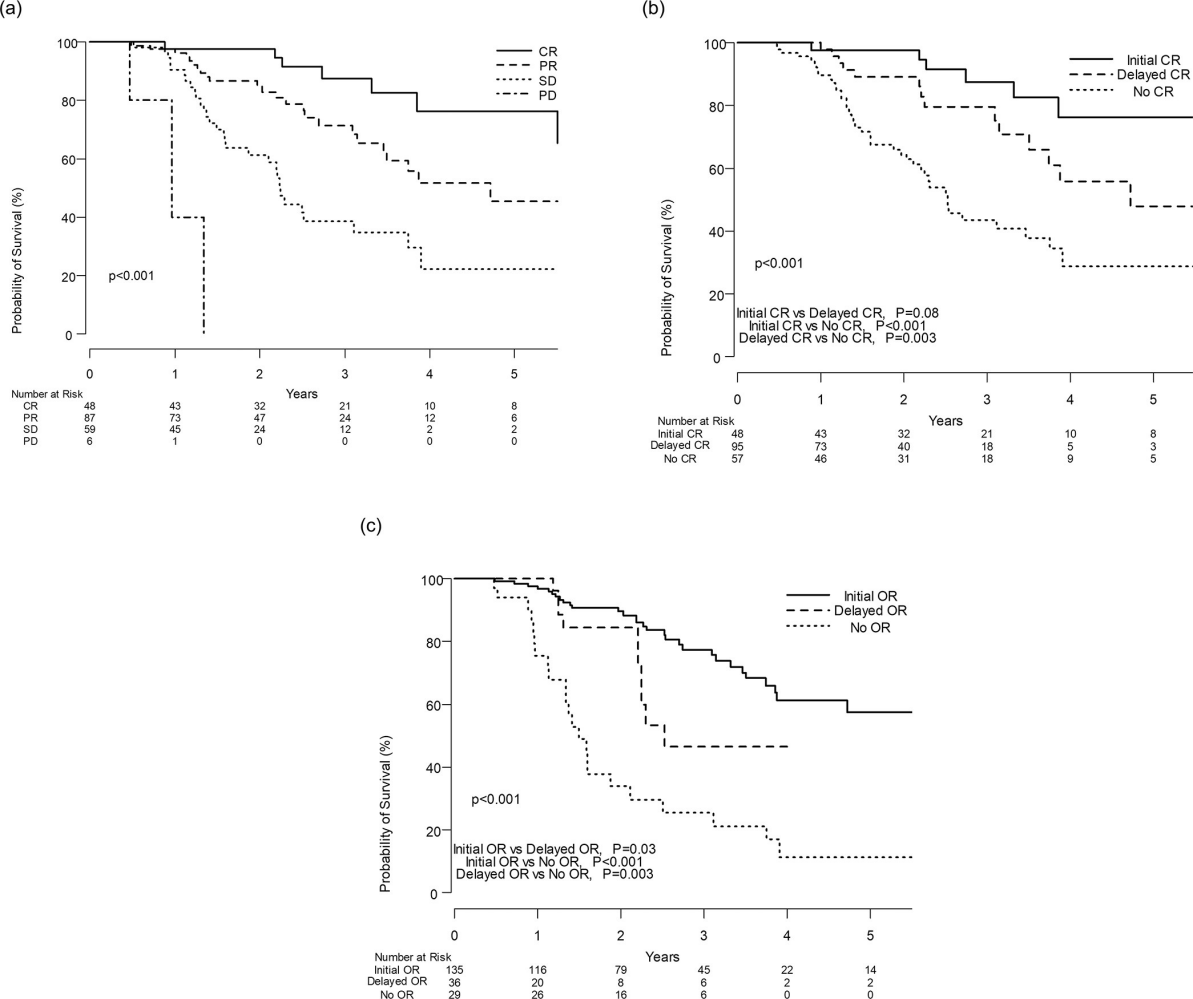
**A.** Kaplan-Meier analysis of overall survival in patients according to their responses to the first TACE session. **B.** Kaplan-Meier analysis of overall survival among patients achieving CR at the first TACE session, patients achieving CR at subsequent session of TACE, and patients not achieving CR. **C.** Kaplan-Meier analysis of overall survival among patients showing objective response in the first TACE session, patients showing objective response in subsequent sessions of TACE, and patients showing no objective response.

The median OS of the study population as a whole was 46.8 months. Although median OS of patients who achieved CR could not be estimated, median OS after the first TACE session in patients who achieved PR, SD, and PD was 56.6, 26.9, and 11.5 months, respectively (Fig 4A). Patients who achieved CR following the first TACE (Initial CR group) showed a better OS compared with those who achieved CR at the subsequent TACE (Delayed CR group) or those who failed to achieve CR (no CR), (Fig 4B). However, the median OS in the initial CR group did not differ significantly from OS in the delayed CR group (P = 0.08). According to time of OR, patients achieving an OR in their first TACE session (Initial OR group) had significantly better OS compared with those achieving OR at the subsequent TACE (Delayed OR group) or those who did not show OR (No OR group), (Fig 4C).

## Discussion

This study demonstrated that failure of TACE procedures to control tumors twice consecutively in patients with intermediate-stage HCC or outside the Milan criteria leads to poor OR to subsequent TACE. These findings were consistent with the concept of TACE refractoriness mentioned in the JSH guidelines.[7] Although repeated TACE is known to worsen liver function, in patients without a clear OR, as presented here, TACE is frequently repeated even when other effective systemic therapies are available for the treatment of HCC. The OPTIMIS study, an international prospective observational study found that, at the time when patients became ineligible for TACE due to poor response, only 47 (9.3%) of a total of 507 patients were switched to sorafenib, with the remaining 460 (90.7%) being selected for continued TACE but not sorafenib in the follow-up period.[18] This result suggests that a significant number of patients with HCC in the clinic are treated with TACE, despite the absence of an adequate indication based on the concept of TACE refractoriness.[18] Therefore, our findings, added to the OPTIMIS study results, clearly show that systemic therapy for HCC after TACE refractoriness is not commonly used in clinical settings and that the time for starting systemic therapy varies widely.

In South Korea, once systemic therapy has been initiated for HCC, the national insurance program does not cover further TACE or other locoregional treatment options. Therefore, patients with HCC tend to continue TACE rather than switching to systemic therapy until tumor progression reaches a stage no longer manageable by TACE. This trend was also observed in a subset of Korean patients enrolled in the OPTIMIS study.[18]

Previous Japanese studies reported that median OS was significantly longer in patients who switched to sorafenib than in those who continued TACE or underwent hepatic artery infusion after TACE refractoriness was confirmed.[19–21] In addition, the time until deterioration of liver function was observed or advanced-stage HCC confirmed was also longer in the group switched to sorafenib than in the group that had continued TACE.[19, 20] In the OPTIMIS study, OS of patients who transitioned to sorafenib at the time of TACE refractoriness tended to be longer than in patients who were maintained on TACE therapy or transitioned at a later stage to sorafenib.[18] In keeping with these studies, we show that a lack of response following two consecutive TACE procedures resulted in poor OR regardless of subsequent repeated TACE sessions. These results confirm the robustness of the concept of TACE refractoriness as defined by treatment guidelines.[6, 7]

In keeping with previous Japanese studies[19, 20, 22] we found a deterioration of liver function in patients that were refractory to TACE. However, the degree of deterioration was not significant in our study, possibly because TACE refractoriness was defined as progression after two sessions of TACE. Subsequent TACE procedures were typically performed within 1–2 months after the first TACE, therefore this relatively short interval may not have been sufficient to detected deterioration of liver function in our patients.

Considering there was no significant difference in survival between the initial CR group and the delayed CR group, we believe that repeated TACE could be justified to achieve CR, resulting in a survival gain even in subsequent TACE sessions. In addition, compared to patients without OR after repeated TACE, patients who initially showed an OR showed significantly better survival, followed by patients who achieved OR in subsequent sessions of TACE. This suggests that when there is an OR to TACE, repeated TACE can be advocated in terms of survival gain, whereas without an OR twice consecutively, then further TACE may be unnecessary and could be avoided.

Studies have proposed subdividing patients with intermediate BCLC B-stage HCC because it consists of a heterogeneous population in terms of tumor size and number.[23, 24] Interestingly, a recent report showed a higher propensity of TACE refractoriness and liver function deterioration in patients with intermediate-stage HCC beyond the Milan and beyond the up-to-7 criteria, defined by the Kinki criteria B2.[22] Similarly, in this study, the mean size of the largest tumor in the 45 patients who were refractory to repeated-TACE was 10.3 cm and 44.4% of those patients had 4 or more tumors. In contrast, patients who responded to TACE had favorable tumor characteristics, defined by the Kinki criteria B1.[22] Similarly, we found that the ORR to first TACE in patients within up-to-7 criteria was significantly higher (91.7%) than that in patients beyond the up-to-7 criteria (28.9%). These characteristics may enable robust predictions about whether patients will show favorable responses to repeated TACE, enabling patients who do not show a favorable response to be more rapidly switched to a systemic therapy rather than continuing ineffective TACE with increased possibility of liver function deterioration.

This study did present some limitations. Firstly, OS was longer than reported in previous studies.[21, 22] This may be due to the high ORR of our study population to TACE given the fact that our center is an experienced, high-volume center, treating thousands of HCC patients per year. Previous randomized trials and observational studies have reported ORRs ranging from 40% to 50% [3, 25] whereas the ORR to TACE in our center was 68% in this study and up to 80% in a previous study reported by our group[11]. Despite this, the median OS of patients refractory to TACE was 15.9 months, in keeping with that of previous studies where median OS were reported between 10.3 and 16.2 months.[18–20] Secondly, only a small number of patients were switched to sorafenib treatment after TACE refractoriness, thus preventing a comparison between the outcomes of these patients versus those who were not switched to systemic therapy. In addition, TACE methods may vary across country or center. Thus, our result may not be extrapolated to centers where different TACE methods use. Finally, this study was performed at a single center and was designed retrospectively. Unlike randomized trials, there may have been a selection bias in the timing and decision to switch therapy to sorafenib. However, randomized trials would be precluded due the rarity of this disease.

## Conclusions

Our findings show that patients who did not achieve OR after two sessions of TACE showed poor OR to subsequent TACE treatment. The availability of multiple effective systemic agents for HCC indicates that patients who become refractory to TACE should be transitioned as soon as possible to other treatment options, taking into consideration the patients’ liver functional reserve and performance status.

## Supporting information

S1 Data (XLSX)

## Abbreviations

AFP: alpha-fetoprotein
AOR: adjusted odds ratio
BCLC: Barcelona Clinic Liver Cancer
CI: confidence interval
CR: complete response
CT: computed tomography
HCC: hepatocellular carcinoma
IQR: interquartile range
JSH: Japanese Society of Hepatology
mRECIST: modified response evaluation criteria in solid tumors
MRI: magnetic resonance imaging
ORR: overall response rate
OS: overall survival
PD: progressive disease
PR: partial response
RT: radiation therapy
SD: stable disease
TACE: transarterial chemoembolization
